# On delirium

**DOI:** 10.4103/2152-7806.76976

**Published:** 2011-02-21

**Authors:** Rebecca A. Stout, Moises F. Gaviria

**Affiliations:** Department of Psychiatry, Clinical Psychologist, Advocate Christ Medical Center, Oak Lawn, Illinois, USA; 1 Professor Emeritus, University of Illinois at Chicago, Director of the Neuropsychiatric Division, Advocate Christ Medical Center, Oak Lawn, Illinois, USA

Delirium represents a common neuropsychiatric syndrome that impacts an estimated 20% of postoperative neurosurgical patients.[6] Unfortunately, the successful identification of this common problem continues to be hampered by the diverse terminology and lack of systematized assessment of delirium. A large number of cases of delirium go undetected leading to increased complications and mortality for our patients. We suggest that a larger number of delirium cases would be identified with use of small measures, that if undertaken consistently, would lead to improvement as has been demonstrated in the literature. We argue that undertaking this task of improved identification should be the pooled effort of all health care professionals involved in the care of the postsurgical patient. Psychiatric treatment services are essential in assisting with the management of this population, however, as members of psychiatry departments are often in the service role of consultants to the medical team, we can only assist when the problem is identified and communicated effectively.

One of the frustrations that impede proper identification of delirium concerns disrupted communication. While delirium is a defined disorder with a definite name, there continues to permeate an unclear and inconsistent language in describing the illness among professionals. It is common, in our experience to have delirium communicated using a host of names, such as ICU psychosis, sun-downers, change in mental status, new-onset dementia, acute psychosis, acute brain syndrome, and so on. As long as this inconsistency in language persists, the problem of undetected delirium will continue.

The confusion of terms also leads to another interesting dilemma of responsibility for treatment of the illness. When terms, such as “psychosis” or “dementia,” are used to describe the delirium, the focus on determining the underlying medical cause can be lost in favor of attempting to find a psychiatric basis for the presentation. Delirium is a psychiatric illness that occurs in the context of underlying toxic, metabolic, structural, infectious, and other medical problems. In other words, before delirium can be adequately managed, it must be viewed as a joint effort between psychiatry and the other medical teams involved in the care of the patient, including neurosurgery.

Another barrier in successful identification is the wide variety of the presentation of delirium in individual patients. The diagnosis of delirium includes common symptoms across persons, including disturbances in arousal, attention, cognition, and perception. However, the phenomenology of these deficits ranges wildly. These differences in presentation at least partially account for the errors in description of the illness described above. Patients with *hyperactive* delirium who are actively hallucinating, combative, and disrupting their own care are much more likely to elicit the attentions of medical personnel and generally be identified as delirious, but also more likely to be described as having a psychiatric illness. In contrast, patients with *hypoactive* delirium face greater risk to go undetected given the absence of more disruptive behaviors and the appearance of greater medical plan compliance despite their significant impairments. Both types of patients are equally likely to suffer the negative medical outcomes associated with prolonged delirium if untreated, highlighting the need for improved assessment of the disorder.

Appropriate assessment of delirium should be routinely performed throughout a patient’s hospitalization, especially for those groups at increased risk, including postsurgical, geriatric, and, chronically ill patients among others. Use of validated assessment tools for delirium appears to be woefully lacking in routine care. Estimates suggest that only 16% of medical units use a specific instrument to assess delirium.[1] Fortunately, there are several user-friendly assessment measures that all health care professionals can be trained easily to use and that take only a few minutes to administer. Measures including the Confusion Assessment Method (CAM) This is the correct measure reference,[3] Mini-Mental State Exam,[2] and others can greatly assist in the quick identification of potential delirium. The CAM method has gained a good deal of attention and has now been empirically validated with a number of medical populations. Psychiatry services can assist in these efforts by taking on the leadership role of training medical staff in appropriate use of these instruments.

Pharmacologic and nonpharmacologic methods are available to assist in the management of delirium. The antipsychotic haloperidol has long been used as a first-line treatment, and more recently, the usefulness of atypical antipsychotics, such as quetiapine and olanzapine,[5,4] have also been examined with some promising results. The medical pros and cons of each of the above-mentioned management tools are well outlined in the expanding medical literature and are too broad to be discussed in much depth here. When available to the medical service, consultation of a psychiatrist to assist with these issues is essential. In addition to these medical interventions, several nonpharmacologic techniques applied to the patient, family, and environment are helpful in the management of delirium. These include frequently reorienting the patient, encouraging the presence of family at the bedside, educating family on symptoms and typical course of delirium, correcting sensory impairments (hearing aids, corrective lenses, and so on), and establishing appropriate day/night signals (shades open during the day, lights dimmed at night), and so on. Undertaking these measures consistently will assist substantially in the management of patients experiencing delirium.

Consistent and repeated education among all medical staff is crucial to increase understanding and successful identification of delirium. Top-down and peer-to-peer pressure to utilize the correct terminology when describing delirium will be the essential first step in improving accurate identification of delirium. Given the prevalence and potential negative consequences of delirium, we remain optimistic that appropriate assessment and treatment of this condition will flow from increased education by members of psychiatry departments.
